# Pilonidal Disease: A Recent Decade of the Disease among Active Duty United States Military Service Members

**DOI:** 10.1093/milmed/usag015

**Published:** 2026-02-03

**Authors:** Dylan M Griffiths, Meg I Robinson, Amber L Dougherty, Laura S Kraemer, Andrew J Macgregor, James D Wallace, Diego A Vicente, Christian S Mcevoy

**Affiliations:** Department of General Surgery, Naval Medical Center San Diego, San Diego, CA 92134, United States; Epidemiology and Data Management Support Department, Naval Health Research Center, San Diego, CA 92106, United States; Leidos, Inc, San Diego, CA 92121, United States; Epidemiology and Data Management Support Department, Naval Health Research Center, San Diego, CA 92106, United States; Leidos, Inc, San Diego, CA 92121, United States; Department of General Surgery, Naval Medical Center San Diego, San Diego, CA 92134, United States; Epidemiology and Data Management Support Department, Naval Health Research Center, San Diego, CA 92106, United States; Department of General Surgery, Naval Medical Center San Diego, San Diego, CA 92134, United States; Uniformed Services University of the Health Sciences, Bethesda, MD 20814, United States; Department of General Surgery, Naval Medical Center San Diego, San Diego, CA 92134, United States; Uniformed Services University of the Health Sciences, Bethesda, MD 20814, United States; Military Population Health Directorate, Naval Health Research Center, San Diego, CA 92106, United States; Department of General Surgery, Naval Medical Center San Diego, San Diego, CA 92134, United States; Uniformed Services University of the Health Sciences, Bethesda, MD 20814, United States

## Abstract

**Introduction:**

Sacrococcygeal pilonidal disease (PD) is a common surgical problem in the U.S. Military. Despite recent advancements, treatment strategies remain heterogenous and incompletely described, particularly among the active duty component in the Military Health System (MHS). This study’s objectives were to identify the burden of PD and describe current healthcare utilization and treatment patterns in the MHS during a recent 10-year study period.

**Materials and Methods:**

The study population included all active duty U.S. service members with at least one PD diagnosis in the MHS Data Repository between January 1, 2013, and December 31, 2022. For each patient, the first PD diagnosis during the study period was the incident case, and a subsequent case was defined as a second PD diagnosis >30 days after the first and categorized as recurrent (procedure performed at incident encounter) or persistent (no procedure performed at incident encounter). Procedures were identified by Current Procedural Terminology codes.

**Results:**

During the study period, 25,039 active duty service members were diagnosed with an incident case of PD. The study population was mostly male, less than 30 years old, enlisted, and in the Army or Navy. The overall incidence rate was 1.7 per 1,000 person-years. Prevalence of recurrence was 44.6% (2,494 of 5,598) and persistence was 50.1% (9,731 of 19,441). Overall, there were 115,387 medical encounters with a PD diagnosis during the study period, and service members in the top 25% for healthcare utilization had ≥11 encounters with a PD diagnosis.

**Conclusions:**

Pilonidal disease imposes a significant burden on the MHS and is a threat to overall force medical readiness. Treatment strategies remain heterogeneous, and prospective studies are needed to refine clinical guidelines.

## INTRODUCTION

Sacrococcygeal pilonidal disease (PD) is the result of a complicated inflammatory process involving repeated trauma to vulnerable skin and soft tissue with debris and hair in the gluteal cleft.^1–6^ It is an acquired disease affecting hirsute adolescents and young adults, most commonly males.^1^^,^^7–9^ PD mostly occurs in the second and third decades of life with an estimated prevalence between 26 and 100 per 100,000 persons worldwide, and some data suggest the disease burden is increasing.^1^^,^^7–9^ Historically, PD has been one of the most common surgical problems encountered among active duty U.S. service members with nearly 80,000 cases reported during World War II.^10^

The presentation of PD ranges from asymptomatic skin pits in the gluteal cleft to painful acute abscesses and cellulitis, which can be characterized by chronic wounds intercalated with purulence, hair, debris, and inflammatory tissue.^1^ Half of all cases present with active infection and drainage, and the location of the wound often results in significant localized pain. Morbidity also includes adverse psychosocial effects, limitations in completion of basic daily activities, and considerable burden on the healthcare system.^11–13^ From 2000 to 2012, the incidence of PD among non-deployed active duty service members was 1.7 per 1,000 person-years, with over 2,000 service members requiring hospitalization and an estimated 20,000 lost duty days.^13^

Treatment strategies employed in civilian and military healthcare settings are similarly heterogenous and recurrence rates are comparable across populations.^1^^,^^10^^,^^14–19^ In general, abscesses and cellulitis may require incision, drainage, and antibiotics. Management is guided by disease presentation, extent, and severity, and may be further influenced by factors such as care setting and provider specialty. Options range from non-operative observation to minimally invasive procedures and complex surgical interventions, including wide excisions with reconstruction or healing by secondary intention.^20^ Preventive measures include improved hygiene, routine hair removal, and laser hair depilation.^1^^,^^20^ Although no consensus exists on an optimal treatment strategy, significant improvements in operative management and secondary prevention have been made in recent years.^17^^,^^21^

Despite these recent advances, there are knowledge gaps in the military. Specifically, there are no recently reported data on the incidence of PD or prevalence of recurrence or persistence among the active duty component. In addition, there has not been an analysis of burden on the Military Health System (MHS). This study aimed to identify the burden of PD in the MHS, including incidence, recurrence, and persistence over a recent 10-year period, and to describe current healthcare utilization and treatment patterns.

## METHODS

### Study Population

The study population for this retrospective analysis was obtained from the Military Health System Medical Data Repository (MDR). Active duty U.S. Military personnel with at least one PD diagnosis between January 1, 2013 and December 31, 2022 (*n *= 25,039) were identified from inpatient and outpatient medical encounters in the MDR using *International Classification of Diseases*, 9^th^ and 10^th^ Revision codes (ICD-9 685.0-.1; ICD-10 L05-.02, L05.9-.92) in any diagnosis position. The inpatient and outpatient medical databases used included the Comprehensive Ambulatory/Professional Encounter Record (CAPER) Basic and Enhanced, MHS GENESIS, TRICARE Encounter Data Institutional (TED-I) and Non-Institutional (TED-NI), Standard Inpatient Data Record (SIDR), and Standard Ambulatory Data Record (SADR).

### Measures

For each service member, their first medical encounter during the study period was considered the incident case of PD. Subsequent PD cases were defined as a medical encounter with a PD diagnosis >30 days later and was categorized as recurrent (procedure performed at incident encounter) or persistent (no procedure performed at incident encounter). The prevalence of recurrence and persistence was calculated using an end date of December 31, 2023, to allow for at least one-year of follow-up for service members with an incident case in 2022. Demographic variables included age, sex, service branch, military rank, and medical setting.

Healthcare utilization was assessed using all inpatient and outpatient medical encounters with a PD diagnosis during the study period, with a limit of one encounter per person per day for analysis. Treatment strategies were identified from Current Procedural Terminology (CPT) codes and grouped by incision and drainage procedures, excision procedures, other, and none. Information on oral antibiotics and opioids prescribed within 3 days of the incident PD encounter were obtained from the Pharmacy Data Transaction Service and reported by procedure status.

### Statistical Analyses

All statistical analyses were performed in Python with code executed in Jupyter Notebook via the Anaconda distribution. The overall PD incidence rate for active duty service members was calculated in 1,000 person-years using denominator data from the Defense Manpower Data Center from September 1, 2013 to August 31, 2022, and the study population was restricted to those dates for this calculation. Descriptive statistics were reported. Chi-square tests compared study population characteristics by incident cases, and prevalence of recurrence or persistence. Healthcare utilization for PD was reported as counts of MHS encounters (median, top 10% [≥90th percentile], and top 25% [≥75th percentile]) with a PD diagnosis. Prevalence of PD recurrence and persistence was calculated as the proportion of service members with a subsequent PD diagnosis >30 days after incident diagnosis who had a procedure at incident encounter (recurrent case) or no procedure (persistent case). Cumulative recurrence and persistence rates were calculated as the time from incident diagnosis to the first recurrent or persistent PD encounter occurring >30 days later.

## RESULTS

Characteristics of the study population are shown in Table 1. During the study period, 25,039 service members were diagnosed with PD. Most were male (86.1%), less than 30 years old (79.2%), enlisted (89.6%), and in the Army (38.0%) or Navy (24.7%). Almost all incident encounters (99.3%) occurred in an outpatient setting. The overall incidence rate of PD was 1.7 per 1,000 person-years. Of the incident cases, 22.4% had a surgical procedure performed at the incident encounter. Overall, 48.8% of the study population had a subsequent diagnosis of PD >30 days after the incident encounter, and the prevalence of recurrence (procedure at incident encounter) was 44.6% (2,494 of 5,598) and persistence (no procedure at incident encounter) was 50.1% (9,731 of 19,441). The distributions of incident cases and rates of recurrence and persistence were statistically different for all covariates (*p*s < .05).

**Table 1.** Study Population Characteristics by Incident Pilonidal Disease (PD) and Prevalence of Recurrence and Persistence

Characteristic	Incident PD			***P*-value** ^a^	Subsequent PD >30 days after incident PD			***P*-value** ^a^
	Total	Procedure	No procedure		Total	Recurrence^b^	Persistence^c^	
Total, *n* (%)	25,039 (100)	5,598 (22.4)	19,441 (77.6)	<.001	12,225 (48.8)	2,494 (44.6)	9,731 (50.1)	<.001
Age, years, *n* (%)				<.001				<.001
<30	19,841 (79.2)	4,681 (83.6)	15,160 (78.0)		9,924 (50.0)	2,029 (44.7)	7,833 (51.7)	
30+	5,198 (20.8)	917 (16.4)	4,281 (22.0)		2,301 (44.3)	403 (43.9)	1,898 (44.3)	
Sex, *n* (%)				<.001				<.001
Male	21,555 (86.1)	4,608 (82.3)	16,947 (87.2)		10,644 (49.4)	2,021 (43.9)	8,623 (50.9)	
Female	3,484 (13.9)	990 (17.7)	2,494 (12.8)		1,581 (45.4)	473 (47.8)	1,108 (44.4)	
Service branch, *n* (%)				<.001				<.001
Navy	6,190 (24.7)	1,521 (27.1)	4,669 (24.0)		3,035 (49.0)	705 (46.4)	2,330 (49.9)	
Marine Corps	4,308 (17.2)	910 (16.3)	3,398 (17.5)		2,103 (48.8)	392 (43.1)	1,711 (50.4)	
Army	9,507 (38.0)	2,256 (40.3)	7,251 (37.3)		4,602 (48.4)	978 (43.1)	3,630 (50.1)	
Air Force	5,034 (20.1)	911 (16.3)	4,123 (21.2)		2,485 (49.7)	425 (46.7)	2,060 (50.0)	
Rank, *n* (%)				<.001				<.001
Enlisted	22,434 (89.6)	5,179 (92.5)	17,255 (88.8)		10,979 (48.9)	2,304 (44.5)	8,675 (50.3)	
Officer/WO	2,605 (10.4)	419 (7.5)	2,186 (11.2)		1,246 (47.8)	190 (45.3)	1,056 (48.3)	
Medical setting, *n* (%)				.027				.017
Outpatient	24,871 (99.3)	5,548 (99.1)	19,323 (99.4)		12,162 (48.9)	2,473 (44.6)	9,689 (50.1)	
Inpatient	168 (0.7)	50 (0.9)	118 (0.6)		63 (37.5)	21 (42.0)	42 (35.6)	

Abbreviations: PD, Pilonidal Disease; WO, Warrant Officer.

a Chi-square tests.

b Calculated by row with the *n* size for Procedure at Incidence PD as the denominator.

c Calculated by row with the *n* size for No Procedure at Incident PD as the denominator.

The distribution of CPT codes by total and incident PD encounters is shown in Table 2. Overall, there were 115,387 medical encounters with a PD diagnosis from 2013 to 2023 and a median of 3.0 encounters per person (range: 1-189). Service members in the top 10% for healthcare utilization had ≥16 encounters with a PD diagnosis, and those in the top 25% had ≥11 encounters during the study period. Of all encounters, 79.3% had no procedures documented, 12.8% had incision and drainage procedures, 8.8% had excision procedures, and <1% had other more complex tissue transfer procedures. Incident encounters followed a similar treatment pattern. Notably, of the 19,441 service members who did not have a procedure at incident encounter, 8,572 (44.1%) had a procedure documented at a subsequent encounter (see Table S1). Incision and drainage procedures had the lowest cumulative recurrence rate (44.4%), followed by excision procedures (46.4%), whereas those with no procedure at the incident encounter had the highest rate of persistence (50.1%). Cumulative recurrence increased rapidly across all procedure groups within the first 5 months after incident encounter (data not shown).

**Table 2.** Distribution of Current Procedural Terminology Codes (CPT) for Pilonidal Disease (PD) and Prevalence of Recurrence or Persistence Among Active Duty U.S. Service Members, 2013-2023

CPT group, code, and description	Procedures performed, *n* (%)^a^		% recurrence or persistence^b^
	Total encounters (*n *= 115,387)	Incident encounters (*n *= 25,039)	
Incision and drainage procedures	14,303 (12.8)	5,247 (21.0)	44.4
10060: Simple or single I&D	4,505 (3.9)	1,202 (4.8)	43.3
10061: Complex or multiple I&D	797 (<1)	245 (1.0)	40.0
10140: I&D of hematoma, seroma, fluid	70 (<1)	2 (<1)	100
20000: Superficial incision of soft tissue abscess	0	0	
20005: Deep/complicated incision of soft tissue abscess	189 (<1)	58 (<1)	53.5
10080: I&D of a pilonidal cyst	6,045 (5.2)	3,385 (13.5)	44.4
10081: I&D of a complicated pilonidal cyst	1,131 (<1)	609 (2.4)	47.3
17999: Unlisted procedure on skin/subcutaneous tissue	1,999 (1.7)	24 (<1)	75.0
Excision procedures	9,459 (8.8)	349 (1.4)	46.4
11770: Excision of a simple pilonidal cyst or sinus	3,908 (3.4)	227 (<1)	48.5
11771: Excision of an extensive pilonidal cyst or sinus	4,236 (3.7)	105 (<1)	44.8
11772: Excision of a complicated pilonidal cyst or sinus	1,978 (1.7)	51 (<1)	58.8
Other procedures	253 (<1)	2 (<1)	100
15576: Under flaps (skin and/or deep tissues) procedures	0	0	
10180: Complex I&D for postoperative wound infection	38 (<1)	2 (<1)	50.0
17380: Electrolysis epilation	215 (<1)	1 (<1)	100.0
No procedures	91,469 (79.3)	19,441 (77.6)	50.1

a Percentages do not total 100% because of multiple procedures within or across procedure groups.

b Calculated as the proportion of patients with a subsequent PD diagnosis >30 days after incident diagnosis who had a procedure at incident encounter (recurrent case) or no procedure at incident encounter (persistent case).

Overall, 31.0% (*n *= 7,762) of service members were prescribed oral antibiotics within 3 days after incident PD encounter and 18.8% (*n *= 4,718) were prescribed opioids (Table 3). Among those with a surgical procedure at incident encounter, 23.1% were prescribed antibiotics and opioids, 22.4% antibiotic only, and 20.2% opioid only. Among those with no surgical procedure at incident encounter, 6.7% were prescribed antibiotics and opioids, 20.1% antibiotic only, and 5.1% opioid only.

**Table 3.** Number and Percent of Antibiotic and Opioid Prescriptions at Incident Pilonidal Disease Encounter by Surgical Procedure Status

Prescriptions, *n* (%)	Total (*n *= 25,039)	Procedure (*n *= 5,598)	No Procedure (*n *= 19,441)
Antibiotic only	5,157 (20.6)	1,256 (22.4)	3,901 (20.1)
Opioid only	2,113 (8.4)	1,129 (20.2)	984 (5.1)
Antibiotic and opioid	2,605 (10.4)	1,295 (23.1)	1,310 (6.7)
None	15,164 (60.6)	1,918 (34.3)	13,246 (68.1)

## DISCUSSION

Pilonidal disease has been a persistent threat to U.S. Military medical readiness since World War II.^13^ Findings from the present study are consistent with previous research and demonstrate that the incidence of PD among active duty U.S. Military service members has been constant at 1.7 per 1,000 person-years over the past two decades.^13^ This analysis also showed that PD often results in high health care utilization, including multiple medical encounters and procedures, and is frequently paired with oral antibiotic and opioid prescriptions. Treatments for the disease were heterogenous, and risk mitigation strategies, such as laser hair depilation,^17^ were rarely utilized. Nearly half the study population had either recurrent or persistent disease, regardless of whether a procedure was performed at incident encounter, and cumulative disease persistence was highest among those treated initially without a procedure.

The incidence of PD among the active duty component is not exclusive to the U.S. Military. Analyses of other military populations have demonstrated comparable incidence rates with 2.4 per 1,000 person-years in a German cohort^22^ and 0.2 per 1,000 person-months in an Israeli cohort.^23^ To further understand the considerable burden of PD in the military, it is important to note that PD incidence in the MHS is similar to that of appendicitis (1.6 per 1,000 person-years), which is a recognized surgical problem in the active duty component.^24^ Altogether, these studies demonstrate that PD is a sizeable problem that adversely affects military populations on a global scale.

The present analysis identified high rates of PD recurrence and persistence, with approximately 49% of cases incurring a subsequent diagnosis of PD >30 days after incident encounter. This is consistent with a previous analysis on the active duty component from 2000 to 2012,^13^ which indicates that improved treatment strategies throughout the MHS are needed. Although there is no downward trend in the incidence or recurrence of PD in civilian and military populations, specific surgical techniques have contributed to a decreased workforce burden in the military.^22^^,^^25^

The present study demonstrated a heterogeneity of treatments for PD across the MHS, yet a relative homogeneity of high recurrence and persistence of PD across treatments. Two decades of data from Israeli military PD-specific clinics showed improved morbidity, less time lost to convalescence, and comparable recurrence when a minimally invasive trephination procedure popularized by Gips et al.^21^ was employed.^23^ In a series published from an Italian civilian hospital, 2,347 patients treated with this surgical technique had a recurrence rate of only 5.8% with a median follow-up of 16 months.^26^ Further, in a meta-analysis, Amorim et al.^27^ pooled 4,286 patients across 13 studies and evaluated PD recurrence after the trephination procedure, and found a similarly low early recurrence rate of 6.5% within 2 years post-procedure and a median return-to-work time after the procedure of a single day. The cumulative recurrence, however, was 38.9% within 2 years after surgery,^27^ which is no worse than the recurrence of PD in this present analysis. Overall, outcomes following the trephination procedure among both civilian and military populations are promising and deserve more study in the U.S. active duty component.^23^^,^^26^^,^^27^ Another treatment approach that has demonstrated promise in reducing PD recurrence is laser hair depilation, yet this analysis found the therapy was rarely documented in the MDR,^17^ and prospective studies are needed to assess the efficacy of this treatment among U.S. service members.

This study had several limitations. Because PD is often a chronic disease that starts in childhood or adolescence, it is difficult to ascertain incidence in a military cohort. Thus, it is possible that an incident case, as defined in this analysis, may not be the first episode of disease experienced by the patient, but rather the first diagnosis captured in the MDR during the study period. Nevertheless, this case definition is consistent with a previous study of PD incidence among the active duty component.^13^ Additionally, service members with PD and associated procedures were identified with ICD-9/10 and CPT codes in the MDR, respectively, which could result in over- or underestimations because of improper coding or data entry errors. Future research is needed to determine the accuracy and completeness of coding in the MDR. Further, differences in case definitions of PD recurrence and persistence throughout the literature render comparisons difficult. However, compared with a prior study of PD in the U.S. Military, which defined recurrence as greater than >365 days after initial encounter, the present study more precisely captured the scope of disease recurrence and persistence in the MHS.^13^^,^^17^ Last, follow-up time was not standardized in this analysis. As such, cases identified early in the study period contributed more follow-up time than those identified later. Although this afforded a more thorough understanding of PD’s burden in the MHS (e.g., healthcare utilization), differences between groups with respect to recurrence or persistence were more challenging to interpret.

Despite these limitations, there are notable strengths. This study provides the most recent estimates of individual service member and MHS-wide burden of PD since 2012 and highlights that this disease continues to be an immediate and prolonged threat to force health, which could have significant impacts on readiness in future warfare. The use of diagnosis codes from the MDR in the analysis presented unique challenges, but is a robust and comprehensive data source in the MHS, which allowed for estimates of PD incidence, recurrence, and persistence, as well as healthcare utilization and treatments. Moreover, this study is the first to explore the relationship between groups of surgical procedures and time to PD recurrence among active duty U.S. service members. Altogether, these findings indicate that current treatment strategies in the MHS are not effective in comprehensively preventing disease recurrence, nor are they consistent with more successful protocolized PD treatment. The implementation of standardized clinical guidelines is needed, and research on recent advancements in preventive care, such as laser hair depilation that is available at most major military treatment facilities equipped with a dermatology clinic, merits further consideration.

## CONCLUSIONS

Pilonidal disease causes significant morbidity in the U.S military, including individual health- and readiness-related consequences, and is a considerable burden on the MHS because of the incidence and chronicity of disease. Pilonidal disease often leads to high healthcare utilization, such as multiple medical encounters and procedures. This suggests there may be additional adverse effects on readiness, including lost duty time. At present, there is no MHS-wide protocol for the treatment of PD and some recognized risk-reduction strategies appear to be underutilized. Forward action against PD in the form of an organized treatment strategy, enhanced prevention efforts, and prospective analyses are warranted to improve the overall force health and readiness.

## Supplementary Material

usag015_Supplementary_Data

## Data Availability

The data underlying this article cannot be shared publicly because of privacy concerns. The data will be shared on reasonable request to the corresponding author.

## INSTITUTIONAL CLEARANCE

Institutional approval granted by the Naval Health Research Center.
