# Supplementary material for: Pilonidal Disease: A Recent Decade of the Disease among Active Duty United States Military Service Members
Source: Mil Med. 2026 Feb 3;191(11-12):e2222–7. doi: 10.1093/milmed/usag015 (PMC13730450; doi:10.1093/milmed/usag015)
Supplement: Supplementary file 1 [file usag015_supplementary_data.docx]

**SUPPLEMENTAL TABLE I.** Distribution of Current Procedural Terminology Codes (CPT) for Pilonidal Disease (PD) Among 8,572 Active-Duty U.S. Service Members with Delayed Treatment,^a^ 2013–2023

| **CPT Group, Code, and Description** | **Procedures Performed, n (%)**  **(N=13,867)** |
| --- | --- |
| Incision and drainage procedures | 5,521 (39.8%) |
| 10060: Simple or single I&D | 1,425 (10.3%) |
| 10061: Complex or multiple I&D | 240 (1.7%) |
| 10140: I&D of hematoma, seroma, fluid | 37 (<1%) |
| 20000: Superficial incision of soft tissue abscess | 0 |
| 20005: Deep/complicated incision of soft tissue abscess | 50 (<1%) |
| 10080: I&D of a pilonidal cyst | 1,889 (13.6%) |
| 10081: I&D of a complicated pilonidal cyst | 371 (2.7%) |
| 17999: Unlisted procedure on skin/subcutaneous tissue | 1,518 (10.9%) |
| Excision procedures | 8,148 (58.8%) |
| 11770: Excision of a simple pilonidal cyst or sinus | 3,049 (22.0%) |
| 11771: Excision of an extensive pilonidal cyst or sinus | 3,469 (25.0%) |
| 11772: Excision of a complicated pilonidal cyst or sinus | 1,632 (11.8%) |
| Other procedures | 198 (1.4%) |
| 15576: Under flaps (skin and/or deep tissues) procedures | 0 |
| 10180: Complex I&D for postoperative wound infection | 22 (<1%) |
| 17380: Electrolysis epilation | 176 (1.3%) |

^a^Procedures performed among service members with CPTs documented at subsequent PD encounters, but not at incident encounter.
